# Squamous cell carcinoma superimposed on necrobiosis lipoidica: a rare complication^⋆^^⋆⋆^

**DOI:** 10.1016/j.abd.2020.03.014

**Published:** 2020-08-21

**Authors:** Maria Emilia Vieira de Souza, Julia Kanaan Recuero, Manoella Freitas Santos, Renan Rangel Bonamigo

**Affiliations:** a Department of Dermatology, Santa Casa de Misericórdia de Porto Alegre, Porto Alegre, RS, Brazil; b Universidade Federal de Ciências da Saúde de Porto Alegre, Porto Alegre, Rio Grande do Sul, Brazil

*Dear Editor,*

Necrobiosis lipoidica (NL) is a chronic idiopathic disease, of a granulomatous nature, which affects the dermis. The lesions are characterized by brownish-yellow plaques, and the most common location is the pre-tibial region. It more often affects females (3:1) and appears, in general, from the third decade of life onwards. There is an association with diabetes mellitus (DM), and microangiopathy is considered an etiopathogenic factor for the condition. Ulceration is a common complication, while reports of the onset of squamous cell carcinoma (SCC) in NL lesions are rare.^1^, ^2^ After reviewing the literature, 16 cases of SCC in NL lesions were retrieved.^3^, ^4^, ^5^

A 62-year-old female patient presented an ulcerated, hyperkeratotic lesion in the NL area, on the right leg, about three months prior, with no history of local trauma. She had been diagnosed with NL in the pre-tibial region of both legs ten years before, with no history of recurrent ulceration. The patient had controlled systemic arterial hypertension and type 2 DM. She used oral hypoglycemic agents, with good control in the last years, and did not present retinopathy or diabetic nephropathy. At the physical examination, the patient presented atrophic bilateral plaques on the pre-tibial region and, on her right leg, a small hyperkeratotic ulcerated plaque over a NL lesion (Fig. 1). The anatomopathological examination showed a moderately differentiated SCC with invasion of the reticular dermis in an NL lesion (Figure 2, Figure 3). No palpable lymphadenomegalies were observed. The SCC was excised and the wound was closed with a graft. The patient evolved well in the immediate and late postoperative period. The anatomopathological examination showed tumor-free margins.

**Figure 1:**
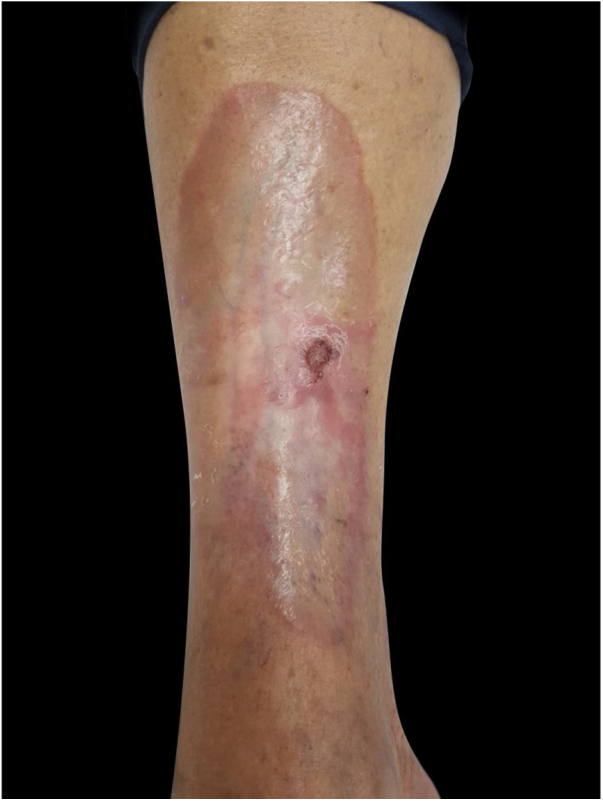
Small hyperkeratotic ulcerated plaque on a necrobiosis lipoidica lesion.

**Figure 2:**
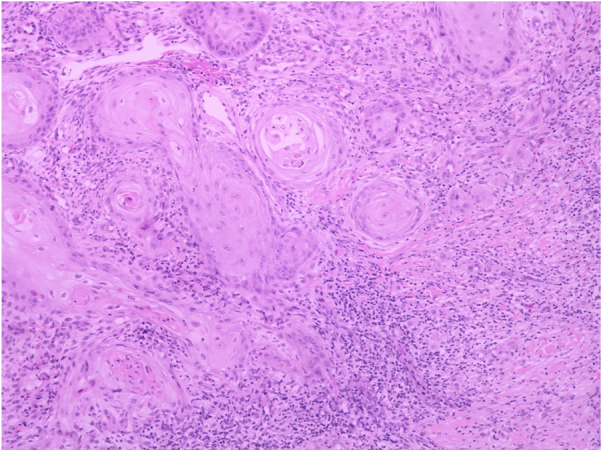
Anatomopathological examination indicating a moderately differentiated squamous cell carcinoma with invasion of the reticular dermis (Hematoxylin & eosin, ×20).

**Figure 3:**
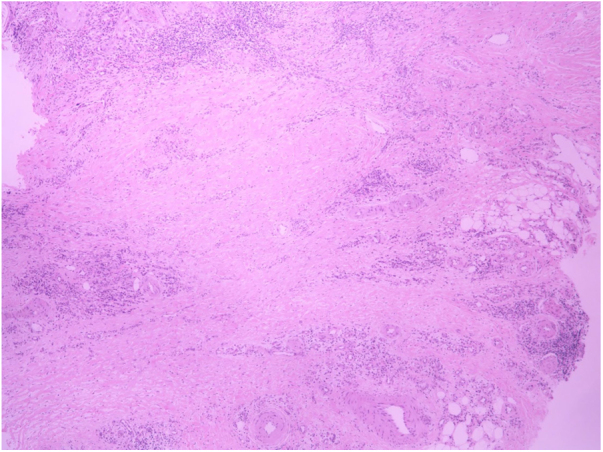
Diffuse areas of collagen degeneration, characteristic of necrobiosis lipoidica (Hematoxylin & eosin, ×5).

NL is a chronic degenerative disease of the dermal connective tissue characterized clinically by yellowish plaques with a narrow granulomatous border, central atrophy, and a tendency to ulceration, most commonly affecting the pre-tibial region of the lower limbs. It is more frequently observed in diabetic patients and, unlike in the reported, most patients also present microvascular complications related to DM, such as nephropathy and retinopathy.

The etiology and pathogenesis of NL are uncertain; it is believed that external trauma, primary vascular disorders, and microangiopathy can contribute to its development, regardless of the presence of DM.^1^

The onset of SCC in areas of ulceration and scarring is well documented in a variety of skin diseases. Ulceration is the main complication of NL, observed in a quarter of patients with this disease. However, despite presenting a chronic course and a tendency to ulceration, the onset of SCC in NL lesions has been seldom reported. It is not clear whether the transformation to SCC is the result of chronic ulceration or of long-standing changes in NL. Risk factors that may be involved in malignant transformation include loss of melanin, which facilitates the lesion, chronic inflammation, and hypoxia.^2^, ^3^ In the present patient, the neoplasm presented as a new ulceration ten years after the diagnosis of the disease, a long period – similarly to other cases described in the literature. However, unlike others, the present case of SCC on an NL lesion did not have a history of recurrent or intractable ulcers.^3^, ^4^, ^5^

Metastases have been described in the literature, and may be related to delayed diagnosis (ulcerations are common in NL, delaying the diagnosis of SCC).^5^ In the case reported, diagnosis and treatment were performed early; the patient is currently being followed-up and is without lymph node metastases or the appearance of new lesions. To the best of the authors’ knowledge, this is the 17^th^ case reported in the international literature.^3^, ^4^, ^5^

The onset of SCC on a NL lesion is very rare. This possibility should be considered in patients with NL with chronic ulcers, as well as in new lesions in patients with NL. Early detection and treatment of SCC associated with NL is of fundamental importance to allow conservative surgical treatment and the best possible clinical outcome.

## Financial support

None declared.

## Authors’ contributions

Maria Emília Vieira de Souza: Approval of the final version of the manuscript; conception and planning of the study; elaboration and writing of the manuscript; critical review of the literature; critical review of the manuscript.

Julia Kanaan Recuero: Approval of the final version of the manuscript; conception and planning of the study; elaboration and writing of the manuscript; obtaining, analyzing, and interpreting the data; intellectual participation in propaedeutic and/or therapeutic conduct of studied cases; critical review of the literature; critical review of the manuscript.

Manoella Freitas Santos: Approval of the final version of the manuscript; conception and planning of the study; elaboration and writing of the manuscript; critical review of the literature; critical review of the manuscript.

Renan Rangel Bonamigo: Approval of the final version of the manuscript, conception and planning of the study, elaboration and writing of the manuscript; obtaining, analyzing, and interpreting the data; effective participation in research orientation; intellectual participation in propaedeutic and/or therapeutic conduct of studied cases; critical review of the literature; critical review of the manuscript.

## Conflicts of interest

None declared.

## References

[1] Sibbald C., Reid S., Alavi A. (2015). Necrobiosis lipoidica. Dermatol Clin..

[2] Boateng B., Hiller D., Albrecht H., Hornstein O. (1993). Cutaneous microcirculation in pretibial necrobiosis lipoidica. Comparative laser doppler flowmetry and oxygen partial pressure determinations in patients and healthy probands. Hautarzt..

[3] Kolovics J., Mattes L.P., Andersen K.E., Beck-Nielsen H. (2015). Squamous cell carcinoma developing in necrobiosis lipoidica in a diabetic patient. Ugeskr Laeger..

[4] Lefkovits Y., Adler A. (2019). Fatal squamous cell carcinoma from necrobiosis lipoidica diabeticorum in a diabetic patient. Endocrinol Diabetes Metab Case Rep..

[5] Uva L., Freitas J., Soares de Almeida L., Vasques H., Moura C., Miguel D. (2015). Squamous cell carcinoma arising in ulcerated necrobiosis lipoidica diabeticorum. Int Wound J..

